# Killer cell immunoglobulin-like receptors (KIR) and human leukocyte antigen-C (HLA-C) allorecognition patterns in women with endometriosis

**DOI:** 10.1038/s41598-020-61702-y

**Published:** 2020-03-17

**Authors:** Ya-Ching Chou, Chi-Huang Chen, Ming-Jer Chen, Ching-Wen Chang, Pi-Hua Chen, Mu-Hsien Yu, Yi-Jen Chen, Eing-Mei Tsai, Peng-Sheng Yang, Shyr-Yeu Lin, Chii-Ruey Tzeng

**Affiliations:** 1 Center for Reproductive Medicine & Sciences, Department of Obstetrics and Gynecology, Taipei Medical University Hospital, Taipei, Taiwan; 2 Department of Obstetrics and Gynecology, School of Medicine, College of Medicine, Taipei Medical University, Taipei, Taiwan; 3 Department of Biological Science and Technology, College of Biological Science and Technology, National Chiao Tung University, Hsinchu, Taiwan; 4 Center for Intelligent Drug Systems and Smart Bio-devices (IDS2B), National Chiao Tung University, Hsinchu, Taiwan; 5 Department of Obstetrics and Gynecology and Women’s Health, Taichung Veterans General Hospital, Taichung, Taiwan; 6 School of Medicine, National Yang-Ming University, Taipei, Taiwan; 7 Graduate Institute of Clinical Medicine, College of Medicine, Taipei Medical University, Taipei, Taiwan; 8 Department of Obstetrics and Gynecology, Shuang Ho Hospital, Taipei Medical University, Taipei, Taiwan; 9 Department of Obstetrics & Gynecology, Tri-Service General Hospital, National Defense Medical Center, Taipei, Taiwan; 10 Department of Obstetrics and Gynecology, Taipei Veterans General Hospital, Taipei, Taiwan; 11 School of Medicine, Institute of Clinical Medicine, National Yang-Ming University, Taipei, Taiwan; 12 General Research Centers of R&D office, Kaohsiung Medical University, Kaohsiung, Taiwan; 13 Division of Reproductive Medicine, Department of Obstetrics and Gynecology, Kaohsiung Medical University Hospital, Kaohsiung, Taiwan

**Keywords:** Genetic association study, Endocrine system and metabolic diseases

## Abstract

Endometriosis shares similarities with several autoimmune diseases. The human leukocyte antigen (*HLA*)*-C* genotype is associated with several human autoimmune diseases. HLA-C is a ligand of killer cell immunoglobulin receptors (KIRs) and is an essential regulator of natural killer cell activity, which is associated with endometriosis progression. Polymorphisms in *HLA-C* and *KIR* affect the activity of NK cells and susceptibility to several diseases. Therefore, we attempted to investigate an association between *HLA-C* genotype and *KIR* polymorphism and the occurrence of endometriosis. We tested the association of certain KIR and HLA-C combinations and the development of endometriosis by characterizing both KIR and HLA-C genes in 147 women with endometriosis and 117 controls. The *HLA-C* genotypes and *KIR* polymorphisms were analyzed via DNA-based method for higher-resolution genotyping. We found that the occurrence of HLA-C*03:03*01 was increased in endometriosis than in control groups. Analysis of various *KIR* haplotypes revealed differences between the endometriosis and control cohorts. The number of *KIR* centromeric A/A haplotypes was increased in the endometriosis group than controls. Moreover, the endometriosis cohort was characterized by reduced number of *KIR2DS2-*positive individuals in the Han Chinese population. Our current findings suggest that the *KIR* and *HLA-C* genotypes are associated with the pathogenesis of endometriosis.

## Introduction

Endometriosis is a chronic gynecological disease with unknown etiology and is characterized by extra-uterine growth of endometrial tissue^1^. Endometriosis affects 6% to 10% of fertile women at the reproductive age and causes severe pelvic pain and infertility^2–4^. Familial and twin studies have reported that genetic factors are associated with the pathogenesis of endometriosis^5–9^. Cell-mediated and humoral immune responses are essential in the pathogenesis of endometriosis, since it is associated with various immunological abnormalities, particularly cell-mediated immunity^10–12^. The activities of cytotoxic T-cells and natural killer (NK) cells are dysregulated in women with endometriosis^13–16^. Increased serum levels of immunoglobulins and autoantibodies, decreased endometrial cell apoptosis, and the production of pro-inflammatory cytokines are observed in endometriosis patients, indicating that endometriosis shares many similarities with autoimmune diseases^11,12,17,18^.

Major histocompatibility complex (MHC) genes, also known as human leukocyte antigen (HLA) genes, are located in chromosome 6p. The genes encoding the human MHC class I (*HLA-A*, *HLA-B*, and *HLA-C*) and class II (*HLA-DR*, *HLA-DQ*, and *HLA-DP*) molecules are the most polymorphic loci in the human genome. *HLA* genes are polymorphic in binding and function in presenting antigen peptides to T-cells. HLA molecules are key factors involved in regulating the specificity of T-cell-mediated immune response in autoimmune and infectious diseases^19–21^.

*HLA* Class I genes encode cell-surface proteins, whose primary functions are to present antigens to cytotoxic CD8^+^ T-cells during the early immune responses^19–21^. Among these, HLA-C plays a minor role in regulating antigen-specific T-cell responses because of low cell surface expression^22^. HLA-C acts as a ligand for killer cell immunoglobulin-like receptors (KIRs), which regulate natural killer (NK) cell-mediated cytotoxicity. The human immunodeficiency virus Nef protein selectively downregulates the production of HLA-A and HLA-B molecules to suppress cytotoxic CD8^+^ T lymphocyte responses^23^. However, Nef maintains stable HLA-C expression levels to inhibit NK cell activation and renders HLA-C as a T-cell restriction element during HIV infection^24^. Importantly, the *HLA-C* genotype has been implicated in several autoimmune diseases, including Graves’ disease, psoriasis, and Crohn’s disease^20,22,23,25,26^.

NK cells are lymphocytes that serve as vital components of the immune system by regulating early responses against infected or transformed cells via cytokine production and direct cytotoxicity^27^. KIRs are a family of membrane glycoproteins expressed by NK cells. KIRs contain two or three extracellular immunoglobulin-like domain molecules (D) with a long (L) or short (S) cytoplasmic tail^28^. The *KIR* gene is located on chromosome 19q13.4 on the leukocyte receptor complex. KIR exhibits activating and inhibitory effects with extensive haplotypic and allelic polymorphisms^29–31^. The 16 KIR genes comprise the following: six genes encoding activating KIR (2DS1-5 and 3DS1), seven genes encoding inhibitory KIR (2DL1-3, 5 and 3DL1-2), KIR2DL4, which can exert both inhibitory and activating activity, and two pseudogenes (2DP1 and 3DP1). Furthermore, *KIR3DL3*, *KIR3DP1*, *KIR2DL4*, and *KIR3DL2* are framework genes and are always present in the genome^32^.

The primary ligands of KIR are HLA-C molecules, which are divided into two groups, namely C1 and C2, based on the amino acid at position 80 [HLA-C C1 groups (HLA-C1), asparagine (N) at position 80: C*01, 03, 07 (01–06), 08, 12 (02, 03, 06), 13, 14, 15:07, 16 (01, 03, 04); HLA-C C2 groups (HLA-C2), lysine (K) at position 80: C*02, 04, 05, 06, 07 (07), 12 (04, 05, 42), 15, 16 (02), 17, 18]^32,33^. The inhibitory receptors KIR2DL2 and KIR2DL3 and activating receptor KIR2DS2 share the same ligand HLA-C1. Activating KIR2DS2 has been reported to be in strong linkage disequilibrium and highly homologous to KIR2DL2. KIR2DL1 and KIR2DS1 bind to HLA-C2^31,34–36^. Combinations of HLA-C with KIR2DS1 and KIR2DS2 have been reported to correlate with the occurrence of autoimmune diseases, leukemia, and inflammatory diseases^37–42^. Polymorphisms in the genes encoding HLA-C and KIR affect NK cells activity and susceptibility to several diseases^31^. *HLA* genotyping is traditionally performed using a serological method. However, detection of the *HLA-C* genotype via serological typing is difficult because of the low HLA-C expression levels at the cell surface, the lack of suitable antisera, and difficulties in protein isolation^22^. Therefore, we employed a DNA-based method for higher-resolution genotyping and investigated the association between the *HLA-C* genotype and endometriosis. Moreover, to analyze the association between certain KIR-HLA-C combinations and the development of endometriosis, we characterized both *KIR* and *HLA-C* gene polymorphisms in 147 women with endometriosis and 117 controls.

## Results

### Frequency distributions of *HLA-C* alleles among endometriosis and control groups

The demographic results of endometriosis and control groups are shown in Table 1. *HLA-C* allele frequencies in endometriosis patients (n = 147, 294 alleles) and control patients (n = 117, 234 alleles) were determined using a sequence-based typing method. The presence of HLA-C*03:03:01 significantly increased the risk of endometriosis with p = 0.0473 [Odds Ratio (OR) = 2.811, 95% confidence interval (CI) = 1.021–7.738] and the statistical power was 43.8% (Table 2). After multiple test analyses using Bonferroni correction, the association was not significant.

**Table 1:** Patient demographic results.

Characteristics	Control n = 117 (%)	Endometriosis n = 147 (%)	p value
Age^a^	38.44 (7.47)	36.08 (6.55)	0.012
BMI^a^, kg/m^2^	23.01 (4.47)	21.58 (3.47)	0.0032
Age of menarche^a^	12.53 (1.21)	12.80 (1.52)	0.5968
Duration of Menstrual cycle^a^	27.95 (4.95)	28.45 (2.97)	0.1392
Dysmenorrhea^b^, n (%)	73 (62.39)	112 (76.19)	0.015

Abbreviations: BMI, body mass index; SD, standard deviation

Mean (SD) for continuous variables. n (%) for discontinuous variables.

^a^Mann-Whitney test.

^b^χ^2^ test.

**Table 2:** Distribution of the *HLA-C* alleles in the endometriosis and control groups.

HLA-C	Control (n = 117, 234 alleles)		Endometriosis (n = 147, 294 alleles)		OR	95% CI	P value
	n	%	n	%			
C*01:02:01	56	23.9	59	20.1	0.798	0.5273 to 1.208	0.2907
C*01:08	0	0.0	1	0.3	2.397	0.09712 to 59.16	1
C*02:02:02:01	0	0.0	2	0.7	4.009	0.1914 to 83.97	0.5055
C*03:02:01	24	10.3	33	11.2	1.106	0.6342 to 1.930	0.7786
C*03:03:01	5	2.1	17	5.8	2.811	1.021 to 7.738	0.0473*
C*03:04:01:01	30	12.8	34	11.6	0.8892	0.5265 to 1.502	0.6886
C*03:04:04	0	0.0	4	1.4	7.265	0.3889 to 135.7	0.1333
C*04:01:01:01	10	4.3	12	4.1	0.9532	0.4043 to 2.247	1
C*04:03	4	1.7	4	1.4	0.7931	0.1962 to 3.207	0.7375
C*06:02:01:01	7	3.0	5	1.7	0.561	0.1757 to 1.792	0.3849
C*07:01:01:01	1	0.4	0	0.0	0.2643	0.01071 to 6.523	0.4432
C*07:02:01:01	39	16.7	61	20.7	1.309	0.8391 to 2.042	0.264
C*07:04:01	1	0.4	1	0.3	0.7952	0.04944 to 12.79	1
C*07:359	1	0.4	0	0.0	0.2643	0.01071 to 6.523	0.4432
C*08:01:01	21	9.0	20	6.8	0.7404	0.3911 to 1.401	0.4138
C*08:03:01	0	0.0	1	0.3	2.397	0.09712 to 59.16	1
C*12:02:02	15	6.4	14	4.8	0.73	0.3449 to 1.545	0.4456
C*12:03:01:01	3	1.3	1	0.3	0.2628	0.02714 to 2.544	0.3269
C*14:02:01	6	2.6	9	3.1	1.2	0.4208 to 3.422	0.7975
C*14:02:03	0	0.0	1	0.3	2.397	0.09712 to 59.16	1
C*15:02:01	8	3.4	14	4.8	1.413	0.5822 to 3.427	0.5151
C*15:05:01	1	0.4	0	0.0	0.2643	0.01071 to 6.523	0.4432
C*16:02:01	1	0.4	0	0.0	0.2643	0.01071 to 6.523	0.4432
C*16:04:01	1	0.4	1	0.3	0.7952	0.04944 to 12.79	1

Each HLA allele has four unique sets denoted by different numbers that are separated by a colon. The first two digits often correspond to the serological antigen; the two digits after the first colon denote the subtypes and order in the genome from the IMGT/HLA Database (www.ebi.ac.uk/imgt/hla/).

The differences in *HLA-C* allele frequencies between the endometriosis and control groups were analyzed using the Fisher’s exact test. Significance was set at a P value < 0.05 and the statistical power was 43.8% calculated by G*Power. OR indicates odds ratio. CI indicates confidence interval.

### Frequency distributions of *HLA-C* group among endometriosis and control groups

We evaluated whether the HLA-C group C1 (HLA-C1) and HLA-C group C2 (HLA-C2) were associated with endometriosis. Analysis revealed no significant differences in HLA-C1 and HLA-C2 frequencies in the endometriosis and control groups (Table 3).

**Table 3:** Distribution of HLA-C ligand in endometriosis and control groups.

HLA-C	Control (n = 117)		Endometriosis (n = 147)		OR	95% CI	p value
	n	%	n	%			
C1	115	98.3	146	99.3	2.539	0.2273 to 28.37	0.5859
C2	29	24.8	36	24.5	0.9842	0.5602 to 1.729	1
C1C1	88	75.2	111	75.5	1.016	0.5783 to 1.785	1
C2C2	2	1.7	1	0.7	0.3938	0.03525 to 4.400	0.5859
C1C2	27	23.1	35	23.8	1.042	0.5869 to 1.849	1

Two-sided Fisher’s exact test was used to estimate the differences between endometriosis and control groups.

n: number of cases with relevant genotypes, OR: odds ratio, CI: confidence interval, Significance was set at a P value < 0.05.

### Frequency distributions of *KIR* genotypes among endometriosis and control groups

Using sequence-specific PCR amplification, we analyzed the *KIR* genotypes in the endometriosis and control groups. The frequencies of the *KIR* genotypes in women with endometriosis and controls and their statistical associations are presented in Table 4. The presence of KIR2DS2 significantly reduced the risk of endometriosis with p = 0.0394 [(OR) = 0.5577, 95% CI = 0.3251–0.9569] and the statistical power was 68.6%. After multiple test analyses using Bonferroni correction, the association was not significant. The two groups showed no significant differences in the remaining *KIR* genotypes.

**Table 4:** Genetic association between *KIR* gene in endometriosis and control groups.

Inhibitory KIR	Control (n = 117)		Endometriosis (n = 147)		OR	95% CI	p value
	n	%	n	%			
KIR2DL1	114	97.4	147	100.0	9.017	0.4608 to 176.5	0.0858
KIR2DL2	37	31.6	31	21.1	0.5778	0.3314 to 1.007	0.0653
KIR2DL3	114	97.4	146	99.3	3.842	0.3942 to 37.45	0.3252
KIR2DL4	117	100.0	147	100.0	—	—	—
KIR2DL5	52	44.4	49	33.3	0.625	0.3788 to 1.031	0.0747
KIR3DL1	115	98.3	145	98.6	1.261	0.1748 to 9.093	1
KIR3DL2	117	100.0	147	100.0	—	—	—
KIR3DL3	117	100.0	147	100.0	—	—	—
**Activating KIR**							
KIR2DS1	39	33.3	46	31.3	0.9109	0.5421 to 1.531	0.7911
KIR2DS2	41	35.0	34	23.1	0.5577	0.3251 to 0.9569	0.0394*
KIR2DS3	29	24.8	25	17.0	0.6218	0.3409 to 1.134	0.1273
KIR2DS4^#^	113	96.6	143	97.3	1.265	0.3096 to 5.173	0.7358
KIR2DS4f	89	76.1	117	79.6	1.227	0.6840 to 2.201	0.5503
KIR2DS4d	60	51.3	76	51.7	1.017	0.6255 to 1.653	1
KIR2DS5	24	20.5	25	17.0	0.7941	0.4264 to 1.479	0.5249
KIR3DS1	44	37.6	48	32.7	0.8044	0.4836 to 1.338	0.4365
**Pseudogene**							
KIR2DP1	114	97.4	147	100.0	9.017	0.4608 to 176.5	0.0858
KIR3DP1	117	100.0	147	100.0	—	—	—

^#^The gene was considered positive if either of the two forms were present. KIR2DS4f - full-length *KIR2DS4* allele variant. KIR2DS4d - deleted *KIR2DS4* allele variant.

Two-sided Fisher’s exact test was used to estimate the differences between endometriosis and control groups.

n: number of cases with relevant genotypes, OR: odds ratio, CI: confidence interval, *versus controls, p < 0.05 and the statistical power was 68.6% calculated by G*Power.

### Frequency distributions of *KIR* haplotypes among endometriosis and control groups

The frequencies of the *KIR* haplotypes in women with endometriosis and controls and their statistical associations are presented in Table 5. The χ^2^ value was calculated by Hardy-Weinberg analysis (χ^2^ > 3.841 showed the subgroup was deviating from the Hardy–Weinberg equilibrium). We revealed differences between the endometriosis and control cohorts. The number of *KIR* centromeric A/A haplotypes was increased in the endometriosis group than controls with p = 0.0394 [(OR) = 1.793, 95% CI = 1.045–3.076] and the statistical power was 68.6%.

**Table 5:** Frequency of centromeric and telomeric *KIR* haplotypes in endometriosis and control groups.

Centromeric	Control (n = 117)		Endometriosis (n = 147)		OR	95% CI	p value
	n	%	n	%			
Cen-A/A	76	65.0	113	76.9	1.793	1.045 to 3.076	0.0394*
Cen-A/B	38	32.5	33	22.4	0.6018	0.3480 to 1.041	0.0713
Cen-B/B	3	2.6	1	0.7	0.2603	0.02670 to 2.537	0.3252
χ^2^	0.47		0.73				
**Telomeric**							
Tel-A/A	71	60.7	96	65.3	1.22	0.7374 to 2.017	0.4442
Tel-A/B	42	35.9	47	32.0	0.8393	0.5026 to 1.402	0.5151
Tel-B/B	4	3.4	4	2.7	0.7902	0.1933 to 3.230	0.7358
χ^2^	0.55		0.39				

Two-sided Fisher’s exact test was used to estimate the differences between endometriosis and control groups.

n: number of cases with relevant genotypes, OR: odds ratio, CI: confidence interval, χ^2^ value was calculated by Hardy-Weinberg analysis (χ^2^ > 3.841 showed the subgroup was deviating from the Hardy–Weinberg equilibrium).

*versus controls, P < 0.05 and the statistical power was 68.6% calculated by G*Power.

### Combinations of KIR and their HLA-C ligands

The frequencies of KIR and their HLA-C ligands were analyzed for their statistical associations with endometriosis (Table 6). HLA-C C1 groups are recognized by KIR2DL2/2DS2 and KIR2DL3, while HLA-C C2 groups are recognized by KIR2DL1/2DS1. Moreover, KIR2DL2/2DL3 also binds to some HLA-C C2 molecules, and KIR2DS4 binds to some HLA-C1 and HLA-C2^31,34–36^. The molecular interactions of KIR gene-HLA-ligands were calculated from the KIR frequency of a total number of HLA ligands. The total number of HLA ligands is shown in Table 3. We calculated the KIR frequency in the combination of different HA ligands. Analysis of various KIR-HLA-C combinations revealed no significant differences between the endometriosis and control cohorts (Table 6). The frequency of *KIR* haplotypes and *HLA-C* combinations also showed no significant differences between the endometriosis and control groups (Table 7).

**Table 6:** Distribution of molecular interactions of *KIR* gene-HLA-ligands in endometriosis and control groups.

Inhibitory KIR-ligand association	Control		Endometriosis		OR	95% CI	p value
	n	%	n	%			
KIR2DL1-HLA-C1/C2	27	100.0	35	100.0	—	—	—
KIR2DL1-HLA-C2/C2	1	50.0	1	100.0	3	0.05947 to 151.3	1
KIR2DL2-HLA-C1/C1	25	28.4	20	18.0	0.5538	0.2834 to 1.083	0.0902
KIR2DL2-HLA-C1/C2	10	37.0	11	31.4	0.7792	0.2704 to 2.245	0.7876
KIR2DL3-HLA-C1/C1	86	97.7	110	99.1	2.558	0.2280 to 28.70	0.5847
KIR2DL3-HLA-C1/C2	26	96.3	35	100.0	4.019	0.1573 to 102.7	0.4355
**Activating KIR-ligand association**							
KIR2DS1-HLA-C1/C2	5	18.5	13	37.1	2.6	0.7918 to 8.538	0.1593
KIR2DS1-HLA-C2/C2	2	100.0	0	0.0	0.0667	0.0008081 to 5.5	0.3333
KIR2DS2-HLA-C1/C1	28	31.8	23	20.7	0.5601	0.2947 to 1.064	0.1016
KIR2DS2-HLA-C1/C2	11	40.7	11	31.4	0.6667	0.2337 to 1.902	0.5932

The molecular interactions of KIR gene-HLA-ligands were shown in the frequency of the KIR gene of HLA-ligands, which was calculated from the KIR frequency of the total number of HLA ligands. The total number of HLA ligands is shown in Table 3. Two-sided Fisher’s exact test was used to estimate the differences between endometriosis and control groups. n: number of cases with relevant genotypes, OR: odds ratio, CI: confidence interval.

**Table 7:** Distribution of molecular interactions of *KIR* haplotypes-HLA-ligands in endometriosis and control groups.

KIR haplotypes	HLA-C genotypes	Control		Endometriosis		OR	95% CI	P value
		n	%	n	%			
Cen-A/A	C1/C1	60	68.2	88	79.3	1.786	0.9397 to 3.393	0.1016
Cen-A/A	C1/C2	16	59.3	24	68.6	1.5	0.5257 to 4.280	0.5932
Cen-A/A	C2/C2	0	0.0	1	100.0	15	0.1818 to 1238	0.3333
Cen-A/B	C1/C1	26	29.5	22	19.8	0.5895	0.3065 to 1.134	0.1338
Cen-A/B	C1/C2	10	37.0	11	31.4	0.7792	0.2704 to 2.245	0.7876
Cen-A/B	C2/C2	2	100.0	0	0.0	0.0667	0.0008081 to 5.5	0.3333
Cen-B/B	C1/C1	2	2.3	1	0.9	0.3909	0.03484 to 4.385	0.5847
Cen-B/B	C1/C2	1	3.7	0	0.0	0.2488	0.009738 to 6.358	0.4355
Cen-B/B	C2/C2	0	0.0	0	0.0	—	—	—
Tel-A/A	C1/C1	50	56.8	74	66.7	1.52	0.8529 to 2.709	0.1853
Tel-A/A	C1/C2	21	77.8	21	60.0	0.4286	0.1382 to 1.329	0.1758
Tel-A/A	C2/C2	0	0.0	1	100.0	15	0.1818 to 1238	0.3333
Tel-A/B	C1/C1	35	39.8	33	29.7	0.6407	0.3551 to 1.156	0.1755
Tel-A/B	C1/C2	6	22.2	14	40.0	2.333	0.7523 to 7.237	0.1758
Tel-A/B	C2/C2	1	50.0	0	0.0	0.3333	0.0066 to 16.82	1
Tel-B/B	C1/C1	3	3.4	4	3.6	1.059	0.2307 to 4.863	1
Tel-B/B	C1/C2	0	0.0	0	0.0	—	—	—
Tel-B/B	C2/C2	1	50.0	0	0.0	0.3333	0.0066 to 16.82	1

The molecular interactions of KIR haplotypes-HLA-ligands were shown in the frequency of KIR haplotypes of HLA-ligands, which was calculated from the KIR haplotypes frequency of the total number of HLA ligands. The total number of HLA ligands is shown in Table 3.

Two-sided Fisher’s exact test was used to estimate the differences between endometriosis and control groups.

n: number of cases with relevant genotypes, OR: odds ratio, CI: confidence interval.

## Discussion

Several factors are involved in the pathogenesis of endometriosis including genetic, neuroendocrine, and immunological factors^43–45^. Abnormal immune responses are recognized in endometriosis patients, including excessive inflammatory cytokine secretion, autoantibody production, and NK cell regulation^17,18^. Endometriosis shares similar characteristics with autoimmune diseases^11,12,17,18^. HLA-C affects viral infections and is implicated in several human autoimmune diseases^22^. HLA-C*06:02 is associated with severe early-onset psoriasis. HLA-C *12:02 was found to be associated with increased susceptibility to Crohn’s disease^20^. HLA-C*03 restricts the cytotoxic CD8^+^ T-cell responses during the Epstein-Barr virus and human immunodeficiency virus infections, as well as during co-infection with the influenza virus and the Sendai virus. Herein, we analyzed the associations between *HLA-C* alleles and endometriosis. Consequently, the presence of HLA-C*03:03:01 increased the risk of endometriosis in Asian women (Table 2). However, after multiple test analyses using Bonferroni correction, the association was not significant.

Previous studies reported no association between *HLA* genotypes and endometriosis in Caucasian women with endometriosis and controls, as assessed by serological typing^46–48^. A serological study showed increased frequencies of the HLA-B*54 and HLA-C*07 alleles in Japanese patients with endometriosis^49^. In a recent study, PCR-restriction fragment length polymorphism analysis revealed that the HLA-DRB1*14:03 and HLA-DQB1*03:01 alleles are associated with endometriosis in Japanese women^50,51^. Another study reported an association between the HLA-A*24, HLA-B*07:02, HLA-C*07:02, and HLA-DRB1*01:01 haplotypes and endometriosis in Japanese women^52^. A previous study showed that *HLA-DRB1* alleles were not associated with endometriosis in Polish women^53^. A literature search identified similar reports from China, which showed an association between endometriosis and the HLA-B*46, HLA-DRB1*15, and HLA-DQA1*0401 alleles^54–56^. The reasons underlying the discrepancies observed among these studies are unclear; however, the results may have been influenced by differences in the ethnicities of the women in the study cohorts and the differences in the detection methods.

The frequency of *KIR3DS1* was significantly lower in endometriosis patients compared to control patients^57^. Moreover, the protective effect of the *KIR2DS5* gene was observed in endometriosis patients^58^. Moreover, Nowak *et al*. showed that the protective effect of *KIR2DS5* was present only in the women who harbored the *HLA-C C2* group^59^. Our current findings revealed that a lower proportion of endometriosis groups, which was characterized by the presence of activating KIR2DS2 compared to the control groups (Table 4). Previous studies have shown that KIR2DL2 is in the linkage disequilibrium with KIR2DS2, which caused the relative activation of KIR receptor, which is responsible for the loss of recognition of HLA-C^60–62^. The different ethnic populations showed different values of *KIR* polymorphisms in elucidating genetic relationships among human populations^63^. Moreover, *HLA* genotyping is traditionally performed using a serological method. Therefore, these discrepancies can be influenced by ethnic or assay methods as they do for discrepancies among *HLA* alleles.

NK cell activity has been reported to be a crucial factor in the recognition and lysis of endometrial cells. NK cell activity and quantity were found to be controversial in women with endometriosis relative to controls^15,64–68^. The observed increase in the proportion of CD158a^+^ (KIR2DL1) NK cells in the peripheral blood and peritoneal fluid in endometriosis patients suggested reduced NK cell cytotoxicity in endometriosis^69^. Moreover, the decrease in the proportion of NK cells facilitates endometrial cell invasion and persistent growth in endometriosis^70^. Maeda *et al*. demonstrated that the increase in KIR2DL1 levels in women with pelvic endometriosis can inhibit NK cell activity^66^. However, the activation of chronic NK cell activation can also play a role in endometriosis^71^. The upregulation of KIR2DS1 expression in peritoneal fluid has been detected in women with endometriosis^72^. Recently, bioinformatic analysis showed that KIR2DS2 expression is upregulated in the secretory phase of endometrium in women with endometriosis relative to control women^73^. Thus, the regulation of NK cell activity is a complex process and can affect the pathogenesis of endometriosis^74^. The final activation status of functional NK cells depends on the homeostasis of all NK cell activation/inhibitory receptors and the corresponding ligands. Then, NK cells are regulated in endometrium in women with endometriosis. Further studies will be worth elucidating the functional relevance of the presence of these receptors and ligand proteins in the endometrium. The limitation of this study is that only the stage III or stage IV endometriosis patients were enrolled to investigate the genetic associations. Thus, our results did not show any association between genes and severity of disease. Further clinically relevant studies on the severity of disease and genetic associations are required.

Our current findings demonstrated the association between *KIR* polymorphisms and *HLA-C* genotypes with endometriosis in women. It is the first study addressing *KIR* polymorphisms and *HLA-C* genotypes of women with stage III or IV endometriosis in Han Chinese women. The results suggested that *HLA-C* and *KIR* genotypes influence the susceptibility for endometriosis. Further studies should investigate the role of NK cells in the pathogenesis of endometriosis.

## Methods

### Patients and controls

Han Chinese women were categorized into the endometriosis (n = 147) and control (n = 117) groups. Endometriosis was diagnosed via laparoscopic examination and confirmed via histological assessment. A total of 147 women were classified under stage III or IV endometriosis in accordance with the Revised American Society for Reproductive Medicine Classification. Women in the control group underwent benign gynecological surgery and showed no evidence of endometriosis including myoma, teratoma, serous cystadenoma, ovarian cyst, ovarian stroma, dermoid cyst, mucinous cystadenoma, paratubal cyst, follicular cyst, simple cyst, hydrosalpinx, corpus luteum cyst, fibrous adhesion, and struma ovarii. Considering that autoimmune disorders are associated with *HLA-C* alleles and *KIR* genotypes, the exclusion criteria comprised autoimmune disorders. The protocol was approved by the Institutional Review Board of the Taipei Medical University Hospital, and all participants have informed consent. All experiments were performed in accordance with relevant guidelines and regulations.

### DNA extraction and *HLA-C* and *KIR* genotype analysis

Genomic DNA was extracted using a DNA whole-blood kit following the manufacturer’s instructions (Kurabo Industries, Osaka, Japan). *HLA-C* was genotyped using an HLAssure™ SE sequence-based typing kit (TBG Biotechnology Corp, Queensland, Australia), which was designed to determine *HLA-C* alleles via polymerase chain reaction (PCR) amplification using a sequence-based typing method. Sequence data were processed using allele-typing software (AccuType™) to identify the *HLA-C* alleles. The genotypes of the *KIR* genes were analyzed using the Lifecodes KIR-sequence-specific oligonucleotide (SSO) typing kit (Immucor Transplant Diagnostics, Inc., Stamford, USA) to identify the *KIR* loci amplified in the sample. The presence or absence of the 16 *KIR* genes was determined using 20 different oligonucleotide probes targeting known *KIR* genes (*KIR3DL3* as positive control, *KIR2DL1*, KIR2DL2*001-3/5, KIR2DL2*004, *KIR2DL3*, *KIR2DL4*, *KIR2DL5*, *KIR2DP1*, *KIR3DL1*, *KIR3DL2*, *KIR2DS1*, *KIR2DS2*, *KIR2DS3*, KIR2DS4*whole exon 4, KIR2DS4*whole exon 5, KIR2DS4*-deleted exon 5, *KIR2DS5*, *KIR3DS1*, KIR3DS1*049N, and *KIR3DP1*). The amplicons were analyzed on a Luminex instrument according to the manufacturer’s instructions. The characteristics of full-length and truncated forms of KIR2DS4 were determined using the following three probes; probe 45: KIR2DS4*all full-length, probe 175: 2DS4*full-length Exon 5, and probe 234: 2DS4*deletion Exon 5. *KIR* genes are divided into centromeric and telomeric haplotypes^75^. In short, centromeric A/A haplotypes contained *KIR2DL3* but not with *KIR2DL2* and/or *KIR2DS2*, centromeric A/B haplotypes contained *KIR2DL3* with *KIR2DL2* and/or *KIR2DS2*, and centromeric B/B haplotypes contained *KIR2DL2* and/or *KIR2DS2* but not *KIR2DL3*. Meanwhile, telomeric A/A haplotypes contained *KIR3DL1* and *KIR2DS4* but not *KIR3DS1* or *KIR2DS1*, telomeric A/B haplotypes contained *KIR3DL1* and *KIR2DS4* with *KIR3DS1* and/or *KIR2DS1*, and telomeric B/B haplotypes lacked *KIR3DL1* and/or *KIR2DS4*^76^.

### Statistical analyses

*HLA-C* allele frequencies, the genotypes of the *KIR* genes and KIR-HLA-C pair frequency in endometriosis patients and control women were compared using the Fisher’s exact test. Odds ratios (ORs) and 95% confidence intervals (CIs) were calculated using the GraphPad Prism software (California, USA). P value <0.05 was considered statistically significant. Multiple tests were analyzed by the Bonferroni correction using the GraphPad Prism software. The normality was analyzed by the Kolmogorov–Smirnov test using IBM SPSS statistics version 22 (New York, USA). The continuous variables of patient demographic results were analyzed by the Mann–Whitney test using the GraphPad Prism software. The discontinuous variable of dysmenorrhea was analyzed by χ^2^ test using the GraphPad Prism software. The statistical power was analyzed by the G*Power version 3.1.9.4^77^. The χ^2^ value was calculated by Hardy–Weinberg analysis (χ^2^ > 3.841 showed the subgroup was deviating from the Hardy–Weinberg equilibrium).
